# Comparing the reliability of the single leg squat test using two, three, and four category ordinal rating scales

**DOI:** 10.7717/peerj.20218

**Published:** 2025-10-15

**Authors:** Yongni Zhang, Yifan Liu, Zhicheng Pan, Hui Gao, RobRoy L Martin, Xinwei Huang

**Affiliations:** 1 Duquesne-China Health Institute, Duquesne University, Pittsburgh, PA, United States of America; 2 Department of Physical Therapy, Yangzhi Affiliated Rehabilitation Hospital of Tongji University, Shanghai, China; 3 Department of Physical Therapy Rehabilitation Science, and Athletic Training, University of Kansas Medical Center, Kansas, United States of America; 4 UPMC Center for Sports Medicine, Pittsburgh, PA, United States of America; 5 Department of Physical Therapy, Duquesne University, Pittsburgh, PA, United States of America; 6 Department of Rehabilitation Therapy, Yangzhi Affiliated Rehabilitation Hospital of Tongji University, Shanghai, China

**Keywords:** Visual assessment, Inter-rater reliability, Intra-rater reliability, Functional performance test

## Abstract

**Background:**

The single-leg squat test (SLST) is supported by evidence for reliability and validity across various visual rating methods, but the optimal number of ordinal categories for assessing movement quality remains unclear. The study aims to determine the most informative and reliable number of ordinal scoring categories for visually rating the SLST.

**Methods:**

A total of 58 subjects with lower extremity injuries participated. A single therapist rated the SLST with a 1-week interval to establish intra-rater reliability. Two therapists independently rated the SLST at the same time point to establish inter-rater reliability. Two-, three-, and four-category ordinal scales were simultaneously used to evaluate SLST performance in rating the components of trunk deviation, hip adduction, and lower extremity internal rotation. Reliability was assessed using unweighted kappa (κ) values.

**Results:**

The overall intra-rater reliability was κ = 0.60, 0.35, and 0.20, with inter-rater reliability being κ = 0.60, 0.61, and 0.33 for the two-, three-, and four-category scales, respectively. When specifically looking at the components of trunk deviation, hip adduction, and lower extremity internal rotation, intra-rater reliability was moderate to good for two-category scales (κ = 0.47–0.65), fair to good for three-category scales (κ = 0.3–0.7), and fair to good for four-category scales (κ = 0.36–0.65). Inter-rater reliability for the three components was good to excellent (κ = 0.65–0.86) for the two-category, good to excellent (κ = 0.69–0.86) for the three-category, and fair to excellent (κ = 0.55–0.76) for the four-category.

**Conclusion:**

When applied to specific movement components, the three-category ordinal scale demonstrated the best balance between detailed assessment and reliability for visually rating trunk deviation, hip adduction, and lower extremity internal rotation during the SLST in patients with lower extremity injuries.

## Background

The evaluation for movement system dysfunction can be important for both assessing musculoskeletal injuries (Martin et al., 2023). Functional performance tests are widely used in the assessment of movement system dysfunction, offering information on strength, balance, and neuromuscular control during functional activities (Kivlan & Martin, 2012; Martin et al., 2023; Powell, Jensen & Johnson, 2018). The single-leg squat test (SLS) is a functional movement test widely used in clinical practice to visually assess lower extremity movement quality (Lewis et al., 2015; McGovern et al., 2018; Ressman, Grooten & Rasmussen Barr, 2019). The visual assessment of the single-leg squat test (SLST) has generally been performed using a multiple-segmental approach, which considers the kinetic chain from the foot to the trunk (Ressman, Grooten & Rasmussen Barr, 2019; Ressman, Grooten & Rasmussen-Barr, 2021). While there is evidence for reliability and validity using a variety of methods to score the SLST, the optimal visual rating method is currently unknown (Gomes et al., 2023; Ressman, Grooten & Rasmussen Barr, 2019).

Visual SLST assessment is widely used in clinical settings due to its ease of application and ability to effectively evaluate functional movement (Nae et al., 2017; Ressman, Grooten & Rasmussen Barr, 2019). Studies support the SLST as a valid test for individuals with hip arthritis (Lenzlinger-Asprion et al., 2017), non-arthritic intra-articular hip conditions (Gomes et al., 2025; McGovern et al., 2019; McGovern et al., 2018), greater trochanteric pain syndrome (Ferrer-Peña et al., 2020), anterior cruciate ligament reconstruction (Hall et al., 2015; Madhavan & Shields, 2011), knee arthritis (Nv et al., 2017), and patellofemoral pain (Crossley et al., 2011; Hansen, Lundgaard-Nielsen & Henriksen, 2021; Herrington, 2014; Levinger, Gilleard & Sprogis, 2006; Nakagawa et al., 2012; Willson & Davis, 2008). Additionally, SLST performance is associated with trunk, hip abductor, and hip internal rotator strength (Barker-Davies et al., 2018; Boudreau et al., 2009; Claiborne et al., 2006; Hollman et al., 2014; Kivlan & Martin, 2012; McGovern et al., 2020; Nakagawa et al., 2012; Stickler, Finley & Gulgin, 2015). Despite its popularity and supporting evidence for validity, the intra- and inter-rater reliability of visual SLST assessment varies from poor to excellent (Ressman, Grooten & Rasmussen Barr, 2019). Adequate reliability is needed to accurately interpret functional test performance. Specifically, intra-rater reliability evaluates the consistency of a single rater over multiple testing sessions, while inter-rater reliability assesses the agreement level among different raters evaluating the same individual (Fleiss, Levin & Paik, 2004).

The current literature has not yet identified the optimal method for visual rating of the SLST. A meta-analysis by Ressman, Grooten & Rasmussen Barr (2019) identified more than 10 methods that have been used for visually rating SLST movement, with two to 10 ordinal categories. Across these ordinal scales reliability assessed using kappa ranged from 0.23 to 0.86 for inter-rater reliability and 0.35 to 0.94 for intra-rater reliability (McGovern et al., 2019; Ressman, Grooten & Rasmussen Barr, 2019; Whatman, Toomey & Emery, 2021; Whelan et al., 2019). Direct comparisons of these results are challenging because studies involved individuals with different types of injuries and used varied protocols. Typically, ordinal scales with fewer categories demonstrate higher reliability but provide less information. In contrast, scales with more categories provide more detailed information, but face greater challenges in maintaining acceptable reliability. For example, a two-category scale may rate performance as simply “pass” or “fail,” whereas a three-category scale may distinguish between “normal,” “moderate deviation,” and “severe deviation,”. Therefore, there is a need to determine the optimal number of ordinal categories that should be used for visually rating movement quality with the SLST.

The aim of this study was to determine the number of ordinal scoring categories that would be most informative in describing performance with reliable results for visually rating the SLST. This objective was accomplished by comparing two, three, and four-category ordinal scales in the visual rating of the SLST within the same population over the same testing period. It is hypothesized the four-category ordinal scales would be most informative in describing performance with reliable results.

## Materials & Methods

### Patients or participants

This cross-sectional study evaluated the inter-rater reliability and intra-rater reliability of three different ordinal rating scales for the SLST. A total of 58 subjects diagnosed with lower extremity injuries participated in this study, with twenty-nine individuals in the inter-rater reliability and twenty-nine in the intra-rater reliability. All participants were recruited through flyers on campus and provided written informed consent prior to their inclusion in the study. All identifiable information were removed from the datasets. The Ethics Committee of the Duquesne University approved the study (Protocol ID:2022/02/14). The study’s inclusion criteria included individuals aged 18 to 45 years with a lower extremity injury (involving the hip, knee, foot, or ankle) diagnosed within the past 6 months. Exclusion criteria were individuals who had undergone recent surgery with weight-bearing restrictions, those with neurological disorders, and/or those incapable of performing the SLST.

### Procedures

Individuals completed two self-reported outcome measures: the Lower Extremity Functional Scale (LEFS) and the University of California at Los Angeles (UCLA) Activity Scale. The LEFS includes 20 questions that assess an individual’s ability to perform daily activities, rated on a 4- category scale ranging from “unable to perform” to “no difficulty”, with total scores ranging from 0 to 80 (Binkley et al., 1999). The LEFS has been shown to have appropriate psychometric properties for measuring lower extremity function (Mehta et al., 2016; Zhang et al., 2025a; Zhang et al., 2025b; Zhang, Zang & Martin, 2025c). The UCLA Activity Scale is supported by appropriate psychometric properties for assessing physical activity levels (Naal, Impellizzeri & Leunig, 2009; Rolfson et al., 2016; Terwee et al., 2011).

The SLST was performed using methods similar to those described by McGovern et al. (2019) and McGovern et al. (2018) with subjects receiving verbal and visual instructions. In summary the individual stood barefoot, with their feet at shoulder-width apart and arms relaxed by their sides. Individuals positioned the foot of the affected leg along the long axis of a “T” shape on the ground. The second metatarsal of the foot was aligned perpendicularly to the stem of the “T,” without contacting the line. Individuals then shifted into a single leg stance on the involved leg, bending the non-stance leg at the knee to a 90-degree angle and keeping the thigh vertical to the stance leg. Individuals were instructed to keep a straight trunk while squatting to a depth where the line in front of their toes was no longer visible (approximately 60° knee flexion). Prior to the actual testing, individuals performed three practice trials of the SLST on the test leg. Following these practice trials, they were asked to perform three consecutive SLSTs. During the SLST process, individuals needed to maintain a balanced and controlled motion, performing the squat at a rate of approximately one squat every two seconds.

For intra-rater reliability, one physical therapist rated individual’s SLST performance at baseline and 1 week. For inter-rater reliability, two physical therapists with 35 and 11 years of experience, independently and concurrently rated SLST performance. SLST was rated for trunk deviation, hip adduction, and lower extremity internal rotation using three ordinal rating forms: a 4-point, 3-point, and 2-point scale (Appendix). Trunk deviations were rated by assessing movements into forward flexion, lateral flexion, and/or rotation, with the movement showing the largest deviation among the three being recorded as the final score for trunk overall deviation. Hip adduction was rated by measuring the vertical displacement of the non–weight-bearing anterior superior iliac spine (ASIS). Lower extremity internal rotation was rated based on tibial tuberosity alignment relative to the second toe. Overall SLST performance score was defined by summing the scores from these three segments. Detailed scoring criteria for each rating form are provided in the Appendix. Before data collection sessions, therapists participated in a 15-minute practice session. During this practice session, the therapists used the ordinal scales to assess the SLST on volunteers with the criteria detailed in the instructions (Appendix 1). Therapists rated SLST performance based on the overall performance observed across the three repetitions of the SLST.

### Statistical analyses

Inter-rater reliability was calculated using the visual assessment data provided by the two therapists. Intra-rater reliability was calculated using the visual assessment data provided by baseline and 1 week later. Unweighted κ (kappa) tests were used to compare agreement between tests for categorical variables including trunk deviation, hip adduction, lower extremity internal rotation, and overall performance. The strength of the agreement is classified as follows: poor (<0.20), fair (0.21–0.40), moderate (0.41–0.60), good (0.61–0.80), and excellent (0.81–1.00) (McHugh, 2012). All statistical analyses were conducted using SPSS Version 29 (IBM; Armonk, NY, USA).

## Results

Table 1 presents demographic information for the subjects. Table 2 presents the inter-rater reliability results for trunk deviation, hip adduction, lower extremity internal rotation, and overall score assessed using ordinal scales with two, three, and four categories. The overall score inter-rater reliability was κ = 0.6 for two-category, κ = 0.61 for three-category, and κ = 0.33 for four-category scales. The overall score intra-rater reliability was as follows: for two-category scales, κ = 0.6 at 1-week intervals; for three-category scales, κ = 0.35 at 1-week intervals; and for four-category scales, κ = 0.2 at 1-week intervals. When specifically looking at trunk deviation, hip adduction, and lower extremity internal rotation, inter-rater reliability was good to excellent (κ = 0.6−0.83) for two-category, good to excellent (κ = 0.69−0.82) for three-category and fair to good (κ = 0.55−0.76) for four-category. Intra-rater reliability over a 1-week interval was moderate to good for two-category scales (κ = 0.47–0.65), fair to good for three-category scales (κ = 0.3–0.7), and fair to good for four-category scales (κ = 0.36–0.65).

**Table 1:** Demographic information for subjects.

Variable	Inter-rater reliability	Intra-rater reliability
*N*	29	29
Age	23.1 years (SD = 3.2)	25.4 years (SD = 5.4)
Height	169.7 cm (SD = 7)	171 cm (SD = 8.8)
Weight	66.9 (SD = 9.9)	70.8 (SD = 18.6)
Female	18 (62%	16 (55.2%)
Male	11 (38%)	13 (44.8)
LEFS	66.2 (SD = 3.6)	65.6 (SD = 4.4)
Right leg involved	19 (65.6%)	13 (44.8%)
Left leg involved	10 (34.4%)	16 (55.2%)
UCLA level		
4–6	13 (44.8%)	10 (34.5%)
8–10	16 (55.2%)	19 (65.5%)
Region of injury		
Hip	4 (13.6%)	3 (12.2%)
Knee	17 (58.4%)	12 (41.4%)
Foot and ankle	16 (55%)	14 (48.2%)

**Table 2:** Evidence of inter-rater and intra-rater reliability for the ordinal scales single leg squat test.

Dimensions	Categories	Inter-rater	Intra-rater
Trunk overall deviation	2	0.83	0.65
	3	0.82	0.67
	4	0.55	0.45
Hip adduction	2	0.65	0.58
	3	0.69	0.7
	4	0.59	0.65
Lower extremity internal rotation	2	0.83	0.47
	3	0.8	0.3
	4	0.79	0.36
Overall performance	2	0.6	0.6
	3	0.61	0.35
	4	0.33	0.2

## Discussion

This study identified evidence supporting the inter-rater and intra-rater reliability of using two, three, and four category ordinal scales to rate the SLST for trunk deviation, hip adduction, and lower extremity internal rotation in patients with lower extremity musculoskeletal injuries. The findings did not support the initial hypothesis that the four-category scale would provide the most informative and reliable ratings, as it showed lower reliability than the two- and three-category scales. Given that both the three- and four-category scales demonstrated low intra-rater reliability for overall scoring, they are not recommended for use in composite SLST assessments. However, when rating specific movement components, the three-category scale demonstrated acceptable reliability and greater clinical interpretability. The three-category ordinal scale matched the two-category scale in inter-rater reliability across all three components, and in intra-rater reliability for the trunk and hip components, while demonstrating higher reliability than the four-category scale in nearly all comparisons. The only exception was intra-rater reliability for lower extremity internal rotation, where the kappa value was slightly lower than two-category scale, but still comparable to that of the four-category scale. Given that the three-category scale offers more information than the two-category scale, the results of this study support the three-category ordinal rating scale as the optimal visual assessment tool for SLST in clinical practice for patients with lower extremity injuries.

This study suggests that a three-category classification is the best approach for the visual assessment of the SLST, effectively balancing detailed evaluation with high inter-rater reliability. Although previous studies have reported the reliability of different SLST rating methods, this study uniquely evaluated inter-rater reliability of two, three, and four categories of ordinal scales simultaneously in the same patient group and at the same time. The three-category ordinal scale demonstrated equivalent inter-rater reliability to the two-category scale across the three segments and total score (κ = 0.6−0.83 *vs* 0.61−0.82), while exceeding the four-category scale in rating the SLST (κ = 0.61−0.82 *vs* 0.33−0.79). In addition, the three-category ordinal scale demonstrated similar 1-week intra-rater reliability to the two and four category ordinal scale across the three segments and total score (κ = 0.3−0.7 *vs* 0.47−0.65) and exceeding the four-category scale in rating the SLST (0.3−0.7 *vs* 0.2−0.65). Capturing patient change is important for evaluating the effectiveness of intervention aimed at improving strength, balance, and neuromuscular control. The two-category scale, offering a simple ‘pass’ or ‘failure’ assessment, shows excellent inter-rater reliability but lacks the nuance to capture enough information to identify more subtle changes in a patient’s progress with the SLST. However, the three-category scale provides clinicians with three distinct ordinal levels of performance, as defined by “normal”, “moderate” and “severe” deviation, allowing for more precise monitoring of SLST change. While the four-category scale may be able to identify more subtle changes, it had lower inter-rater reliability, which may affect the consistency of SLST assessments leading to variations in test interpretation.

This current study found fair intra-rater reliability at a 1-week interval for three-category ordinal scales used to rate the SLST overall, with kappa values of 0.35. In comparison, three studies using the same category ordinal scales and time interval reported higher intra-rater reliability than the current study (Crossley et al., 2011; Lenzlinger-Asprion et al., 2017; Whelan et al., 2019). Crossley et al. (2011), Lenzlinger-Asprion et al. (2017) and Whelan et al. (2019) reported moderate to good intra-rater reliability for overall SLST performance, with kappa values ranging from 0.53 to 0.74. The difference in outcomes could be attributed to the current study using real-time visual assessment of SLST, whereas the previous studies used recorded video for assessment. The study found poor intra-rater reliability at a 1-week interval for four-category ordinal scales used to rate the SLST overall, with kappa values of 0.2. Three studies using four-category ordinal scales reported varying intra-rater reliability compared to the current study, with kappa values ranging from 0.35 to 0.94 and time intervals spanning 4 to 10 weeks (Chmielewski et al., 2007; Poulsen & James, 2011; Weir et al., 2010). These differences could stem from variations in rating criteria and time intervals.

Regarding inter-rater reliability, this study found good to excellent reliability for using two-category ordinal scales to rate the SLST in dimensions of trunk deviation, hip adduction, and lower extremity internal rotation, with kappa values ranging from 0.65 to 0.83. In the dimensions of hip adduction and lower extremity rotation, the current study aligns with the findings of Whatman, Toomey & Emery (2021) and Ageberg et al. (2010), who reported good to excellent inter-rater reliability for two-category scales, with kappa values of 0.55 for hip adduction and 0.92–0.93 for lower extremity rotation. The results of this current study also align with the findings of McGovern et al. (2019), who reported moderate to excellent interrater reliability, with kappa values ranging from 0.6 to 0.83, for categories: trunk, pelvis, hip, knee, and depth of squat. A notable difference is that McGovern et al. (2019) included depth of squat as rating category. In the current study, the overall inter-rater reliability for using two categories was good, with a kappa value of 0.65, which is lower than the excellent interrater reliability (ICC3,1=0.93) found by McGovern et al. (2019). This discrepancy arises from the different methodologies. McGovern et al. (2019) calculated interrater reliability based on the total number of deviations per repetition, whereas our study calculated it based on the total scores of each testing category.

Using the three-category ordinal scales to rate the SLST in dimensions of trunk deviation, hip adduction, and lower extremity internal rotation, this study demonstrated good to excellent inter-rater reliability with kappa values ranging from 0.69 to 0.8. The overall inter-rater reliability for using these scales was moderate, with kappa value of 0.61, aligning with the findings of Frohm et al. (2012), Kaukinen et al. (2017) and McKeown et al. (2014). These studies also reported moderate overall interrater reliability for three- category scales with ICC and kappa values between 0.52 and 0.58. In contrast, this current study found fair to excellent inter-rater reliability for four-category ordinal scales in rating the SLST in dimensions of trunk deviation, hip adduction, and lower extremity internal rotation, with kappa values ranging from 0.55 to 0.79. The overall interrater reliability for this four-category scale was fair, with a kappa value of 0.33. This result is similar to previous studies (Friedrich et al., 2017; Weir et al., 2010). Friedrich et al. (2017), reported poor overall interrater reliability with an ICC of 0.14 while Weir et al. (2010), found moderate reliability with an ICC of 0.41. Chmielewski et al. (2007), found fair to moderate reliability with a kappa value of 0.23−0.53. The discrepancy of inter-rater reliability between three and four categories may be attributed to the increased difficulty for the human eye in distinguishing the four distinct gradations of the four-category ordinal scales compared to the three-category scales.

This study provides evidence supporting the inter-rater and intra-rater reliability of real-time visual assessment for evaluating SLST performance. Unlike previous research that utilized video recordings, this study used real-time assessments, which more closely to the conditions of routine clinical practice. Video-based assessments allow clinicians to pause, replay, and analyze specific movements, which may improve scoring consistency. In contrast, real-time assessments are more dynamic and time-constrained, potentially introducing greater variability. However, they offer a more realistic representation of clinical practice. The findings support the use of a three-category ordinal scale as a feasible and clinically meaningful tool for real-time SLST assessment. The three-category scale provides more clinically informative detail than the two-category scale, while demonstrating higher reliability than the four-category scale. These characteristics make it particularly suitable for real-time visual assessment in routine clinical settings. In order to improve the accuracy of real-time assessments, this study recommends standardized training prior to assessment. In the current study’s protocol, a 15-minute session involved raters practicing real-time scoring on volunteers’ SLST performances, followed by discussion and comparison of their ratings based on the scale’s criteria to ensure consistency in its application. For clinicians with less experience, a longer training period may be necessary to ensure consistent and accurate rating.

### Limitation

The study presents methodological limitations that could impact the generalizability of its results. In this study, two experienced physical therapists conducted preparatory training sessions on the SLST rating criteria prior to data collection. This finding suggests that to obtain reliable results with the assessment method described in this study, similar training for each rater is necessary. In addition, the 1-week interval used for intra-rater reliability may limit the applicability of findings to clinical settings with longer reassessment intervals. Furthermore, the study investigated a young and active demographic with a range of lower extremity injuries which limits its broader relevance across varied demographic groups. The small sample size may limit the statistical power to detect precise reliability estimates, future studies should include larger samples to strengthen the robustness and generalizability of the findings. Moreover, the study conclusions regarding the relative advantages of the three-category scale in rating specific movement components should be interpreted within the context of the study’s methodological conditions. Changes in rater experience, training procedures, population or assessment intervals could potentially alter the reliability outcomes observed.

## Conclusion

The study introduces a method for rating the SLST using ordinal scales with two, three and four categories in patients with lower extremity injuries. The findings show moderate to excellent inter-rater reliability and fair to good intra-rater reliability for trunk overall deviation, hip adduction, and lower extremity internal rotation. Although the three-category scale demonstrated inadequate intra-rater reliability for overall scoring, the version defining performance as “normal,” “moderate,” and “severe” deviation showed acceptable inter-rater and intra-rater reliability when applied to specific movement components of the SLST. The three-category ordinal scale matched the two-category scale in inter-rater reliability across all three components and in intra-rater reliability for the trunk and hip components, which showed higher reliability than the four-category scale in nearly all comparisons. The three-category ordinal rating scale effectively balanced detailed evaluation with good inter-rater and intra-rater reliability in clinical practice for patients with lower extremity injuries. The findings did not support the initial hypothesis that the four-category scale would be the most informative and reliable. Either the two- or three-category scale can be used, with the three-category scale preferred.

##  Supplemental Information

10.7717/peerj.20218/supp-1 Supplemental Information 1 Single leg squat test visual rating instruction

10.7717/peerj.20218/supp-2 Supplemental Information 2 Dataset SLST visual assessment outcome rated by two raters

## Additional Information and Declarations

### Data Availability

The following information was supplied regarding data availability: The raw data is available in the Supplemental Files.
