# Supplementary material for: Comparing the reliability of the single leg squat test using two, three, and four category ordinal rating scales
Source: PeerJ. 2025 Oct 15;13:e20218. doi: 10.7717/peerj.20218 (PMC12535228; doi:10.7717/peerj.20218)
Supplement: Supplemental Information 1 [file peerj-13-20218-s001.pdf]

Rating Form

This rating form is designed to evaluate trunk, hip and lower extremity deviations during single leg squat test. It includes three scales: 4-point (Table S1), 3-point (Table S2), and 2-point (Table S3) rating forms.

Table S1 Four Points Rating Form

	Normal (3)	Mild (2)	Moderate (1)	Extreme (0)
Trunk Overall Deviation<br>(Forward flexion, Lateral flexion, Rotation)				
Hip Adduction				
Lower Extremity Internal Rotation				
Overall performance				

Table S2 Three Points Rating Form

	Normal (2)	Mild/Moderate (1)	Extreme (0)
Trunk Overall Deviation<br>(Forward flexion, Lateral flexion, Rotation)			
Hip Adduction			
Lower extremity internal rotation			
Overall performance			

Table S3 Two Points Rating Form

	Normal (2)	Deviation (0)
Trunk Overall Deviation<br>(Forward flexion, Lateral flexion, Rotation)		
Hip Adduction		
Lower extremity internal rotation		
Overall performance		

The Single Leg Squat Test (SLST) was rated for three movement components: trunk deviation, hip adduction, and lower extremity internal rotation, using 4-point, 3-point, and 2-point ordinal scales (Table S1, S2, S3).

Trunk deviation was assessed in three planes: forward flexion, lateral flexion, and rotation. The largest deviation among these planes was recorded as the trunk overall score.

- **4-point scale (0–3):**
  - Forward flexion: no deviation (3); trunk moves forward <4 cm (2); 4–8 cm (1); >8 cm (0).
  - Lateral flexion: no deviation (3); trunk moves laterally <4° (2); 4–8° (1); >8° (0).
  - Rotation: no deviation (3); trunk rotates <3 cm (2); 3–5 cm (1); >5 cm (0).
- **3-point scale (0–2):**
  - Forward flexion: no deviation (2); <8 cm (1); >8 cm (0).
  - Lateral flexion: no deviation (2); <8° (1); >8° (0).

    - Rotation: no deviation (2); <5 cm (1); >5 cm (0).
- **2-point scale (0–1):**
    - No deviation (1); any observable deviation in flexion, lateral flexion, or rotation (0).

Hip adduction was evaluated by observing the vertical displacement of the non–weight-bearing anterior superior iliac spine (ASIS).

- **4-point scale (0–3):** no drop (3); ASIS drops <2 cm (2); ASIS drops 2–4 cm (1); ASIS drops >4 cm (0).
- **3-point scale (0–2):** no drop (2); ASIS drops <4 cm (1); ASIS drops >4 cm (0).
- **2-point scale (0–1):** ASIS remains level (1); any observable drop >0 cm (0).

Lower extremity internal rotation was assessed based on the alignment of the tibial tuberosity relative to the foot.

- **4-point scale (0–3):** vertical over the 2nd toe (3); between the 2nd and 1st toes (2); between the 1st toe and medial border (1); medial to the foot border (0).
- **3-point scale (0–2):** vertical over the 2nd toe (2); between the 2nd toe and medial border (1); medial to the foot border (0).
- **2-point scale (0–1):** vertical over the 2nd toe (1); medial to the 2nd toe (0).

The overall SLST performance score was calculated as the sum of the trunk (overall), hip, and lower extremity ratings within each scale system.

Single Leg Squat Test Visual Rating Instruction

The instructions provided in this appendix detail the criteria for scoring trunk, hip and lower extremity deviations during single leg squat test using the 4-point (Table S4), 3-point (Table S5), and 2-point (Table S6) rating forms.

Table S4 Four Points Rating Criteria

**4 points**	Normal (3)	Mild (2)		Moderate (1)		Extreme (0)
**Trunk overall Deviation**	Take the worst score above as the score of trunks overall					
Trunk Flexion	No deviation 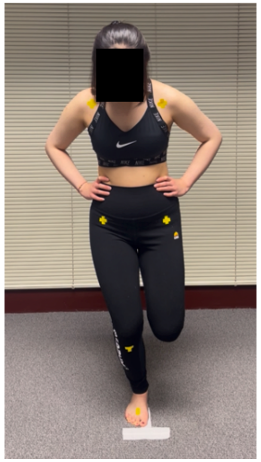	Trunk moves forward <4cm	moves forward 4cm 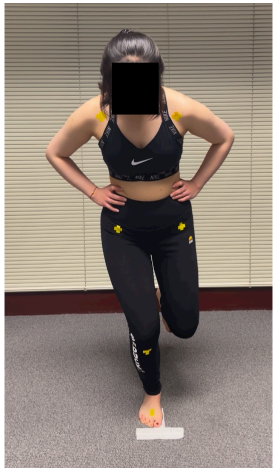	Trunk moves forward 4-8cm.	moves forward 8cm 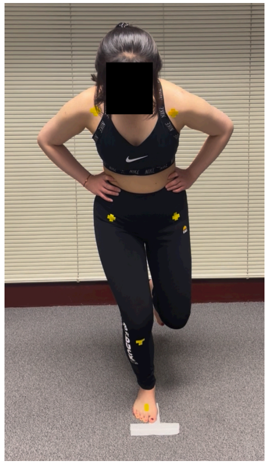	Trunk moves forward >8cm

Trunk Lateral	No deviation	Trunk moves lateral <4 degree	Lateral 4 degree	Trunk moves lateral 4-8 degree	Lateral 8 degree	Trunk moves lateral >8 degree
	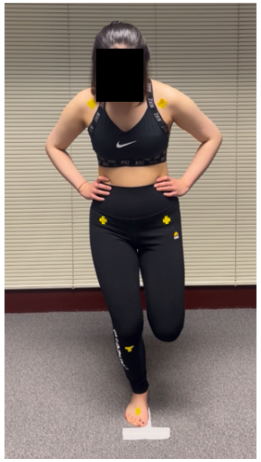		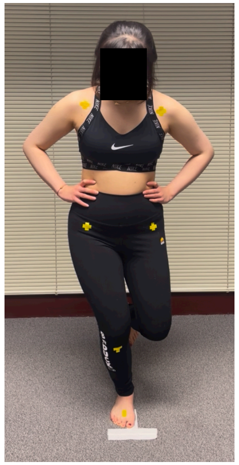		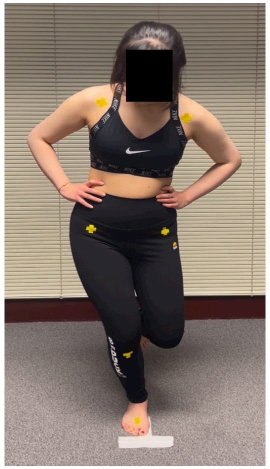	
	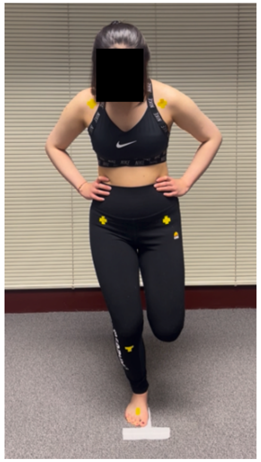		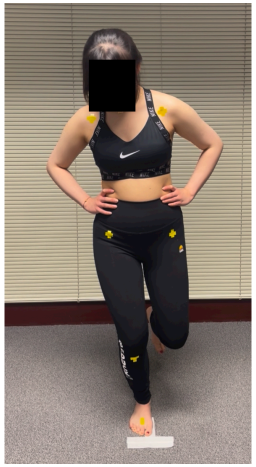		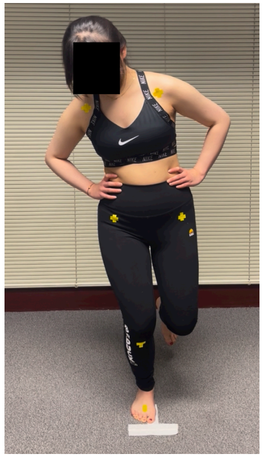	
Trunk Rotation	No deviation		Rotates 3 cm		Rotates 5 cm	

	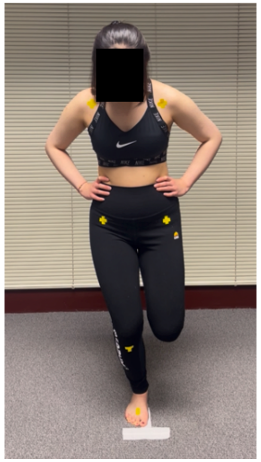<br>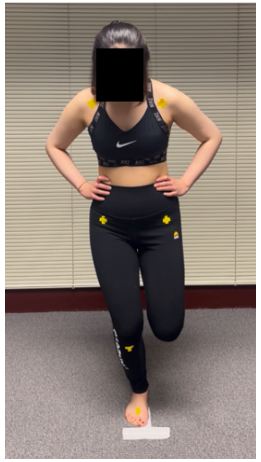	Trunk rotates <3 cm	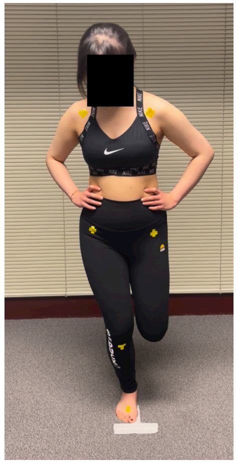<br>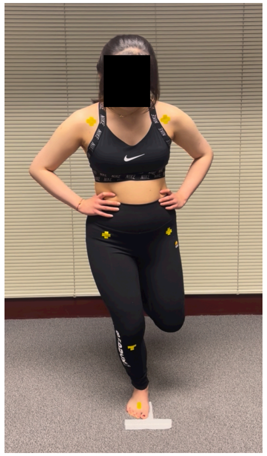	Trunk rotates 3-5 cm	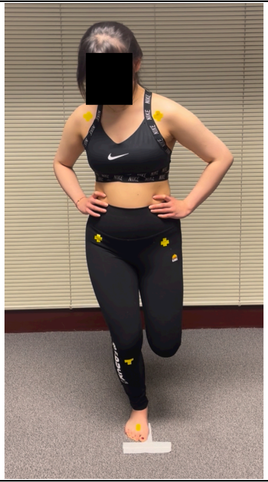<br>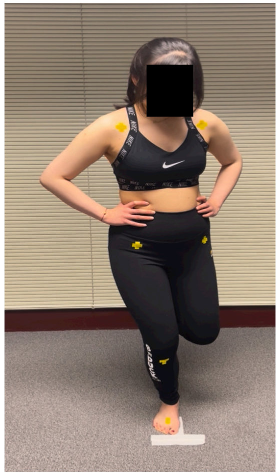	Trunk rotates >5cm
**Hip adduction**	Non-weight bearing side ASIS move down 0 cm	Non-weight bearing side	Non-weight bearing side ASIS down 2cm	Non-weight bearing side	Non-weight bearing side ASIS down 4cm	Non-weight bearing side

	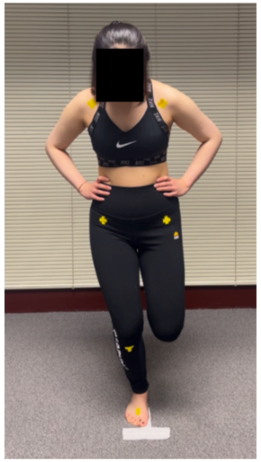	ASIS move down <2cm	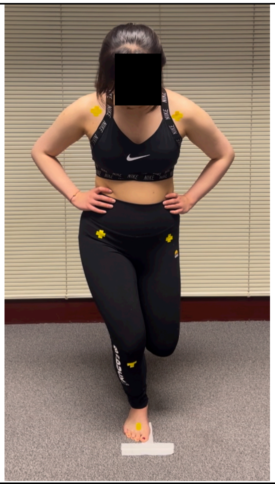	ASIS move down 2-4cm	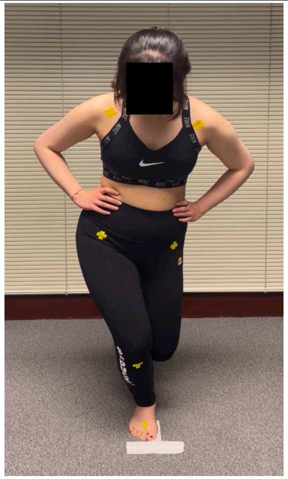	ASIS move down >4cm
**Lower extremity internal Rotation**	Tibial tuberosity vertical line over the 2nd toe 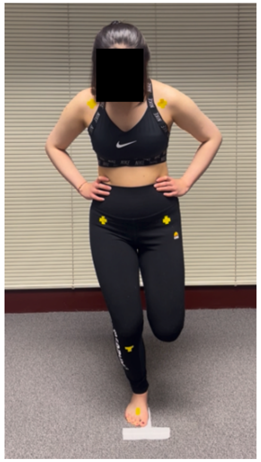	Tibial tuberosity vertical line over 2nd toe- 1st toe	Vertical over 1st toe 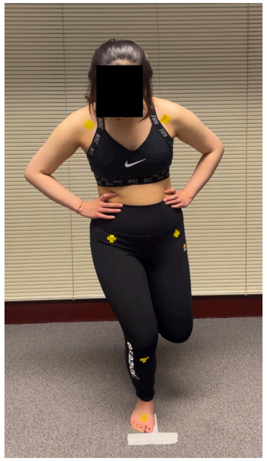	Tibial tuberosity vertical line over 1st toe-Medial border	Vertical over medial border 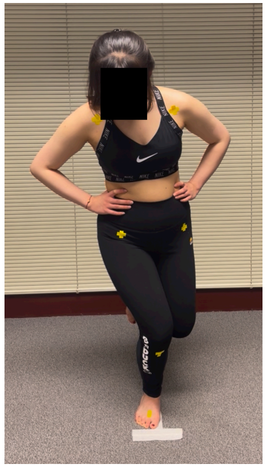	Tibial tuberosity vertical line >medial border

**Overall**	Overall score is the sum of the individual scores for trunk overall deviation, hip adduction, hip internal rotation, and muscle performance.

Table S5 Three Points Rating Criteria

**3 points**	Normal (2)	Mild /Moderate(1)		Extreme (0)
**Trunk overall Deviation**	Take the worst score above as the score of trunks overall			
Trunk Flexion	No deviation<br>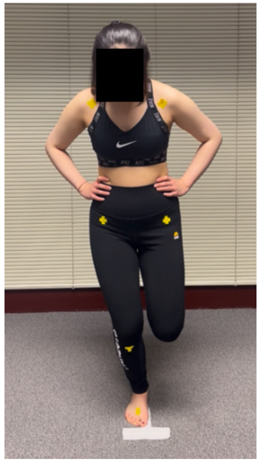	Trunk moves forward <8cm	moves forward 8cm<br>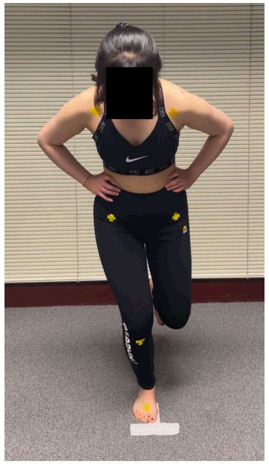	Trunk moves forward >8 cm
Trunk Lateral	No deviation		Lateral 8 degree	

	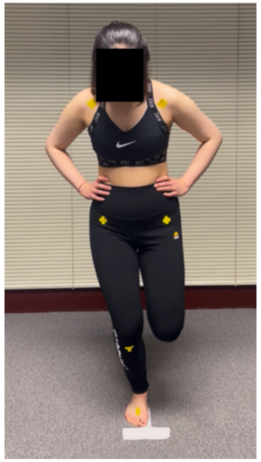<br>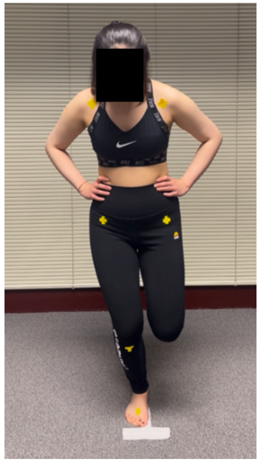	Trunk moves lateral< 8 degree	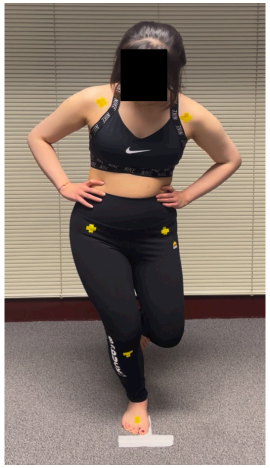<br>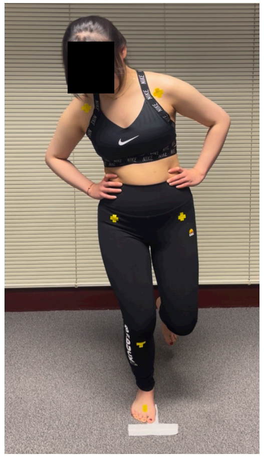	Trunk moves lateral >8 degree
Trunk Rotation	No deviation		Rotates 5 cm	

	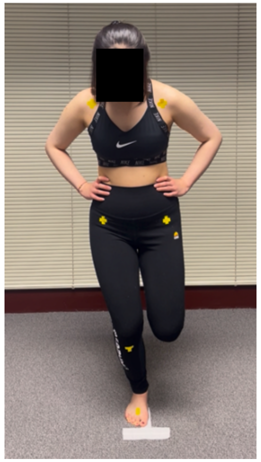	Trunk rotates<5cm	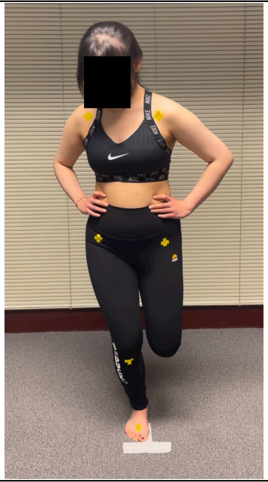	Trunk rotates >5cm
	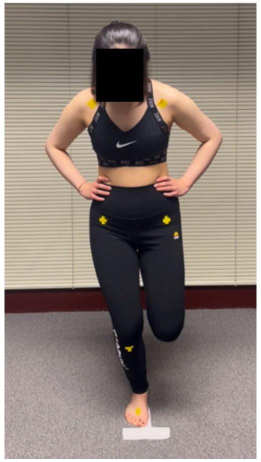		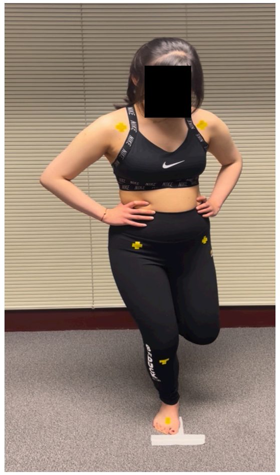	
**Hip adduction**	Non-weight bearing side ASIS move down =0 cm	Non-weight bearing side	ASIS down 4cm	

	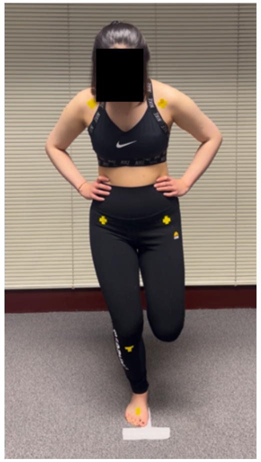	ASIS move down <4cm	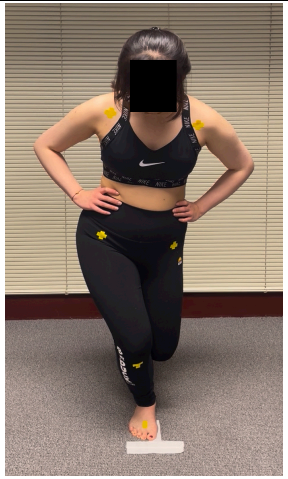	Non-weight bearing side ASIS move down >4cm
**Lower extremity internal Rotation**	Tibial tuberosity vertical line over the 2nd toe<br>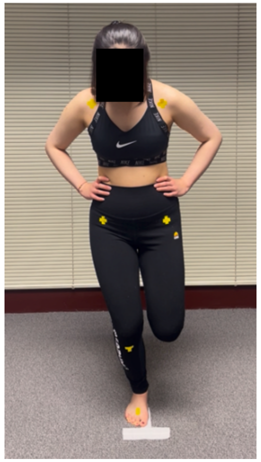	Tibial tuberosity vertical line 2nd toe-Medial border	Vertical over medial border<br>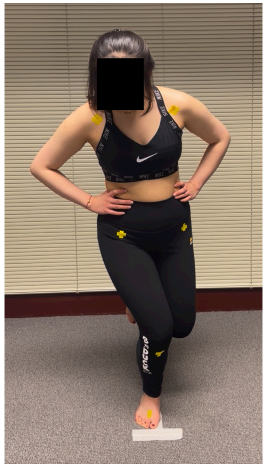	Tibial tuberosity vertical line >medial border

**Overall**	Overall score is the sum of the individual scores for trunk overall deviation, hip adduction, hip internal rotation, and muscle performance.

Table S6 Two Points Rating Criteria

**2 points**	Normal (1)	Deviation (0)
**Trunk overall deviation**	The largest deviation among the following three dimensions being recorded as the final score	
Trunk forward flexion<br>Trunk lateral flexion<br>Trunk rotation	No deviation	Deviation
**Hip adduction**<br>(Non-weight bearing side ASIS)	ASIS move down =0 cm	ASIS move down >0 cm
**Lower extremity internal rotation**<br>(Tibial tuberosity vertical line)	Over the 2nd toe	Medial to the 2nd toe
**Overall performance**	The sum of the individual scores for trunk overall deviation, hip adduction, hip internal rotation, and muscle performance	
